# How primary health care physicians make sick listing decisions: The impact of medical factors and functioning

**DOI:** 10.1186/1471-2296-9-3

**Published:** 2008-01-21

**Authors:** Gunilla Norrmén, Kurt Svärdsudd, Dan KG Andersson

**Affiliations:** 1 Uppsala University, Department of Public Health and Caring Sciences, Family Medicine and Clinical Epidemiology Section, S-75185 Uppsala, Sweden; 2 Örebro University, Department of Clinical Medicine, Family Medicine Research Centre, Box 1613 S-701 16 Örebro, Sweden; 3 National Board of Health and Welfare, Regional Supervising Unit, Box 423, S-701 48 Örebro, Sweden

## Abstract

**Background:**

The decision to issue sickness certification in Sweden for a patient should be based on the physician's assessment of the reduction of the patient's work capacity due to a disease or injury, not on psychosocial factors, in spite of the fact that they are known as risk factors for sickness absence. The aim of this study was to investigate the influence of medical factors and functioning on sick listing probability.

**Methods:**

Four hundred and seventy-four patient-physician consultations, where sick listing could be an option, in general practice in Örebro county, central Sweden, were documented using physician and patient questionnaires. Information sought was the physicians' assessments of causes and consequences of the patients' complaints, potential to recover, diagnoses and prescriptions on sick leave, and the patients' view of their family and work situation and functioning as well as data on the patients' former and present health situation. The outcome measure was whether or not a sickness certificate was issued. Multivariate analyses were performed.

**Results:**

Complaints entirely or mainly somatic as assessed by the physician decreased the risk of sick listing, and complaints resulting in severe limitation of occupational work capacity, as assessed by the patient as well as the physician, increased the risk of sick listing, as did appointments for locomotor complaints. The results for patients with infectious diseases or musculoskeletal diseases were partly similar to those for all diseases.

**Conclusion:**

The strongest predictors for sickness certification were patient's and GP's assessment of reduced work capacity, with a striking concordance between physician and patient on this assessment. When patient's complaints were judged to be non-somatic the risk of sickness certification was enhanced.

## Background

Sickness certification in Sweden should be based on the physician's assessment of a patient having an impaired work capacity owing to disease or injury [1]. However, the law provides poor definitions of the two concepts "disease" and "impaired work capacity". Instead, the legislators refer the physician to a combination of medical assessment, individual experience and social opinions, but provide no definition of disease [2]. This leaves room for individual assessments among doctors and patients on the issue of sick listing and being sick listed. Primary care physicians regard the task of sick listing as one of the most cumbersome in their practise [3]. On the one hand they must do their utmost to maintain a good doctor-patient relationship, on the other hand they also have to comply with the demands of society and refrain from the overuse of public resources.

When impaired work capacity is to be assessed, the International Classification of Functioning, Disability and Health (ICF) [4] offers a tool for describing the level of functioning in a medical context. Usually, a physician's assessment of impaired work capacity is based on a view of the patient's functional impairment in relation to the patient's work tasks, but functional impairment is not always equivalent to reduced work capacity [5]. In most cases, the physician has to rely on the patient's work tasks description. The physician's knowledge of a patient's work demands is thereby second-hand knowledge. The physician's role may be described as assessing the reasonableness of the patient's assessment of his or her impaired work capacity, in order to balance clinical opinion against the intention of the law.

There are some reports on physician-related factors connected to a sick listing decision. Age, postgraduate training and sex have been shown to be related to sick listing [6-9]. In a previous study we found that long experience in family medicine increased the risk of sick leave certification and that physicians working less than full time were more likely to sick list their patients [10].

The main basis for a decision to sick list the patient should be the patient's medical situation. 'Medical' does not necessarily mean the same to the patient as to the physician. The physician may define 'medical' as the field which he/she is familiar with by education and experience, for the patient it might be the field that he/she believes that the physician is an expert on. There are many reports of increased risk of sick listing related to the patient's psychosocial circumstances [11-16]. Reports on a direct relationship between the severity of the medical condition and sick leave are more difficult to find [14,16-22]. Since the legislation does not accept social or labour market related reasons for granting sickness benefits, it is essential to recognise both medical and social reasons for the inability to work [23-26].

There is little literature concerning patients' opinions regarding the influence of their diseases/symptoms on their work capacity [15,17,18,27]. In this study our aim was to explore and compare physician and patient opinions regarding medical factors and functioning and their influence on sick listing.

## Methods

### Setting

The study was performed in 1996 in Örebro county (270,000 residents), central Sweden. GPs in all 26 county council operated primary health care centres (PHCC) and all 11 private family medicine surgeries were invited to participate. Fourteen of the PHCCs and two of the private surgeries took part in the study. Half of the PHCCs represented cities, towns or municipalities with 20,000 inhabitants or more, while the remainder represented smaller municipalities. In this respect the distribution of the non-participating PHCCs was similar.

### Data collection

**Figure 1:**
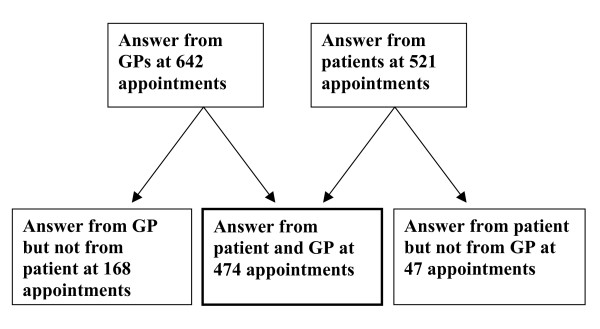
**Study population flow chart**. Distribution of responses from patients and physicians.

The study was performed as a cross-sectional questionnaire study of GPs and consecutive patients, 18–64 years old, who were not already sick listed or retired, came to the practice for whatever reason and were able to fill out a questionnaire. The receptionists were instructed to ask patients in a preset order till up to ten patients per GP were included. The GPs filled out one questionnaire about themselves (Additional file 1), analysed in an earlier paper [10], and one questionnaire about each consultation (Additional file 2). Seventy-three GPs agreed to participate. Six GPs delivered no questionnaires about their patients and two others no questionnaire about themselves. Data delivered by the GPs were available for 642 patients. Patient responses to 521 consultations were returned (Additional file 3). Combined GP and patient responses were available for 474 consultations (Figure 1). Mean physician age was 45 years, 57% were men and 83% were qualified specialists in family medicine. Eleven per cent of the GPs were locums. The majority worked full-time, participated in CME (Continuing Medical Education), patient problem discussion groups and had regular contacts with social insurance officials.

The consultation questionnaire (Additional file 2) included data on patient age, sex, considerations of cause and consequences of the complaints, potential to recover, diagnoses, and issuing of sick notes, which was used as the outcome variable. We made no distinction between part time and full time sick leave. The sickness diagnoses were coded according to the International Classification of Diseases, ninth revision [28]. Three diagnoses could be given. The first two were used in the analyses.

In the patient questionnaire (Additional file 3), data sought were age, sex, native language, education, work and work situation, psychosocial aspects, such as stress and support, and the patients' attitudes to their work, their complaints and consequences of the complaints, information on present and previous health situation, health beliefs, and expectations and outcome of the present consultation. The GP as well as the patient were asked for opinions and attitudes on health, sickness and the welfare system.

The questionnaires were constructed according to our long and comprehensive experience in family medicine in order to cover whatever reason we could figure out possibly to be significant in the sicklisting process. The questionnaires were tested in a pilot study to find out whether the questions were clear-cut and possible to answer. No further validation was made.

The Research Ethics Committee in Örebro approved the study.

### Statistical analysis

The data were analysed using the JMP program package release 5.0 and the SAS software release 6.12 (SAS Institute, Cary, NC, USA). The number of missing values in returned questionnaires was 2.1%. The power of the study to identify a difference of 55% (70.7%–14.6%) in physician assessed reduction of work capacity between those sick-listed and those not sick-listed was 80% with p < 0.005 and a study population of 155 subjects, and more than 99% with the actual study population of 474 subjects. Similar power levels were obtained using the observed difference in patient assessed work capacity.

Possible relationships between answers from the GPs or patients and outcome (issuing of a sick leave certificate) were tested with multivariate logistic regression with backward elimination of non-significant variables, which also provided odds ratios (OR) and confidence intervals (CI).

The responses in the patient questionnaires were divided into two groups of questions. Group A, illustrating the patient's health situation, consisted of questions dealing with previous sick listing pattern, current health and well being, physical leisure time activities and reasons for seeing the GP. Group B, describing functional incapacities, included questions on how the present complaints prevented the patients from working, doing leisure time activities (physical or social), spending time with their families or friends, or getting a good night's sleep.

**Figure 2:**
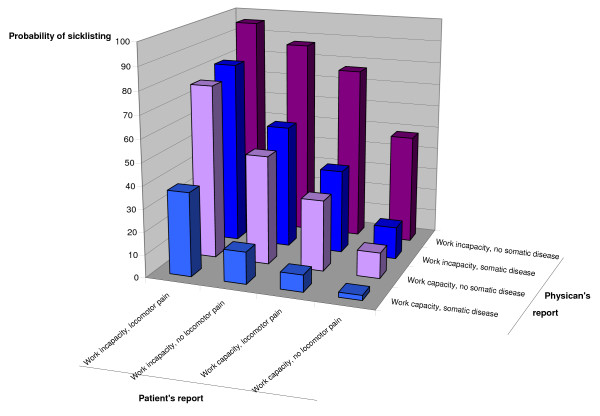
**Combined effects of determinants on sick listing**. Effects on sick listing of patient's report of impairment to work, presence or absence of pain in the locomotor system on the one hand and physician's assessment of patient's impairment to work and whether the condition was primarily somatic or not on the other.

The two groups of answers were first analysed separately. Possible relationships between answers from the GP and patient questionnaires (independent variables) and outcome (issuing of a sick leave certificate, dependent variable) were tested using bivariate and multivariate logistic regression techniques. Statistically significant variables from the two groups were then analysed together with the significant variables from the GP questionnaire. In a final regression model background data as given in Table 1 were introduced in order to see if these variables had any influence on the results, and if so to adjust the results for potential confounding. The logistic regression technique was also used to construct the regression surface in Figure 2.

**Table 1:** Characteristics of 474 patients sick listed or not sick listed in Örebro County primary health care.

	Patients sick listed		Patients not sick listed		
			
	n	mean or %	n	mean or %	p
N	116		358		
Appointment for					
infectious disease^1^	37	30.2	136	38.0	n.s.
musculoskeletal disorder^1^	50	43.1	87	24.4	0.001.
Age, years (SD)		42.7 (11.2)		41.0 (11.8)	n.s.
Women	76	65.5	229	64.0	n.s.
Swedish speaking	109	94.0	341	95.3	n.s.
Education group					n.s.
low	41	35.3	93	26.3	
medium	57	49.1	177	50.0	
high	18	15.5	84	23.7	
Professional status					n.s.
permanently employed	82	70.7	244	68.2	
temporarily employed	18	15.5	49	13.7	
unemployed	8	6.9	30	8.4	
self-employed	7	6.0	21	5.9	

^1 ^One person was reported for both infectious and musculoskeletal disease and is described by both in this table

To estimate the degree to which the determinants could "explain" the variation in sick leave certifications, the correlation coefficient squared (r^2^) was used. Since it is heavily influenced by random variation, the area under the curve of a receiver operator characteristic (ROC) diagram was used as an additional measure [29]. The "degree of explanation" (ROC) was calculated as: (area fraction - 0.5) × 2 × 100.

All tests were two-tailed. During the first two screening stages p-values < 0.05 were accepted as indicating statistical significance. However, in the final analysis model p-values < 0.005 and 99.5% confidence intervals were used to account for multiple testing

## Results

Characteristics of the 116 patients who were sick listed and the 358 patients who were not sick listed as a result of the consultation are shown in Table 1. Of all patients, 173 had infectious diseases and 137 musculoskeletal diseases. Approximately two thirds of the total number were women and about one quarter of the total number were sick listed. The patients who were sick listed were slightly older, mean 42.7 years, than the patients who were not sick listed, mean 41.0 years. The vast majority were Swedish speaking, about 30 per cent had a low educational level, and 75 per cent were permanently employed or self-employed, the remaining were temporarily employed or unemployed. There were no significant differences between patients who were sick listed and not sick listed except that patients with musculoskeletal diseases were sick listed to a significantly greater extent than the whole patient group.

### GP questionnaire (Additional file 2)

Table 2, left panel, shows the GPs' assessments of the patients' complaints and their limitations on work capacity or daily activities. Of sick listed patients, 71% were assessed as having complaints grave enough to limit them severely from occupational work, 53% from physical leisure time activities and 33% from everyday pursuits. The remaining 29% of those who became sick listed were considered to have moderate limitation of work capacity. For those who were not sick listed, the percentage of severe limitation was 15, 21 and 5 respectively. For other types of activities, with fewer reported cases, the percentages of patients considered severely limited by the GP varied from six to 19 among the sick listed patients and one to six among those not sick listed.

**Table 2:** GP assessment of type of complaint, limitations of activities and effects on granting sick leave.

			Multivariate analysis		
			
	Frequencies		All diseases	Infectious diseases	Musculoskeletal diseases
	
	not sick listed n/%	sick listed n/%	OR^1) ^95% CI	OR^1) ^95% CI	OR^1) ^95% CI
Complaints entirely or primarily somatic missing 4	329/92.9	97/83.6	0.24 0.10–0.58	0.04 0.004–0.45	0.16 0.03–0.91
The complaints severely limited the patient in terms of:					
occupational work missing 1	52/14.6	82/70.7	14.15 8.61–23.24	7.81 3.51–17.36	19.11 7.69–47.46
physical leisure time activities missing 22	72/20.8	56/53.3	0.94 0.46–1.94	3.10 0.96–10.07	0.54 0.14–2.14
everyday pursuits missing 20	17/4.9	35/33.0	2.79 1.18–6.55	1.78 0.26–12.27	1.13 0.32–3.96
taking care of his/her children missing 76 ^3)^	10/3.2	17/19.1	0.96 0.25–3.73	7.48 1.24–44.94	0.43 0.06–2.81
sleeping at night missing 21	13/3.8	18/17.0	1.63 0.61–4.36	5.53 0.42–72.96	1.94 0.42–8.92
social leisure time activities missing 24	20/5.8	15/14.4	0.46 0.18–1.15	0.58 0.11–2.99	0.38 0.06–2.37
activities of daily living missing 28	4/1.2	6/5.7	0.58 0.13–2.54	N.A.^2)^	7.57 0.36–157.70
intellectual activities missing 20	5/1.4	6/5.7	1.20 0.24–6.04	1.30 0.15–11.17	N.A.^2)^

^1) ^odds ratio and 95% confidence interval.

^2) ^not applicable owing to non-significance in bivariate analysis.

^3) ^19 of them were 'true missing', i.e. lived with children.

In the bivariate analyses for all diseases statistically significant relation to less sick listing was found for complaints assessed not to be entirely or primarily somatic and to more sick listing for all the limitation variables in Table 2 (data not shown). In multivariate analysis only the variables 'complaints not entirely or primarily somatic', 'severely limited from occupational work' or 'everyday pursuits' remained significantly related to sick listing (Table 2, right panel). When the multivariate regression model was restricted to patients with infectious diseases, the variables 'complaints not entirely or primarily somatic', 'severely limited from occupational work' and 'take care of his/her children' remained significant, and when restricted to patients with musculoskeletal diseases, the same pattern as for infectious diseases could be seen, except for the variable 'take care of his/her children'.

### Patient questionnaire (Additional file 3)

The associations between data in the patient questionnaire and being sick listed are shown in Table 3. In the bivariate analyses in group A for all diseases, differences were significant between patients who were sick listed and who were not sick listed in all variables except the exercise variable, (data not shown). In the multivariate regression model the variables 'sick listing during the last year', 'appointment because of complaints from back, neck, arms/hands or leg/feet', and 'appointment because of tiredness' remained significantly related to sick listing. In the multivariate analysis restricted to patients with infectious diseases, the exercise variable was the only remaining significant variable, and for patients with musculoskeletal diseases 'sick listing during the last year' remained significant.

**Table 3:** Patients' assessment of health and sickness related factors, their effects on daily life and work and effect on sick listing.

			Multivariate analysis		
			
	Frequencies		All diseases	Infectious diseases	Musculoskeletal diseases
	
	not sick listed n/%	sick listed n/%	OR^1) ^95%CI	OR^1) ^95% CI	OR^1) ^95% CI
**Group A questions**					
My general health: missing 8					
Mostly healthy and feel well	240/67.8	64/57.1	1.08 0.64–1.81	N.A.^2)^	N.A^2)^
One or more diseases and seldom or never feel well	7/2.0	9/8.0	2.27 0.74–6.96	N.A.^2)^	N.A.^2)^
Sick listing during the last year missing 3	189/53.1	82/71.3	1.67 1.23–2.26	1.76 0.99–3.14	2.08 1.21–3.55
Exercise weekly or more often to sweatiness or breathless missing 27	170/50.6	49/44.1	N.A^2)^.	0.40 0.18–0.87	N.A.^2)^
Appointment because of: missing 3					
complaints/pain from back, neck, arms/hands or legs/feet	93/26.0	63/54.3	3.49 2.23–5.46	N.A.^2)^	N.A.^2)^
tiredness	24/6.7	17/14.7	2.50 1.25–5.03	2.78 0.94–8.24	N.A.^2)^
anxiety, nervousness, depression, insomnia	15/4.2	11/9.5	2.01 0.83–4.88	N.A.^2)^	N.A.^2)^

**Group B questions**					
My complaints/pain severely limited me from:					
occupational work missing 10	84/26.6	81/81.8	12.43 7.04–21.94	6.35 2.41–16.71	12.98 5.06–33.29
usual leisure activities missing 18	141/41.0	69/61.6	0.56 0.29–1.10	0.98 0.23–4.16	N.A.^2)^
doing daily home work missing 13	69/19.7	56/50.5	1.55 0.84–2.89	1.06 0.40–2.79	1.14 0.41–3.18
sleeping missing 15	62/17.9	37/33.0	1.06 0.43–2.61	N.A.^2)^	2.55 0.93–6.99
seeing friends missing 18	72/20.8	33/30.3	0.55 0.20–1.53	1.02 0.30–3.43	N.A.^2)^
taking care of my children missing 88 ^3)^	25/11.8	14/23.7	0.46 0.15–1.37	N.A.^2)^	N.A.^2)^
taking good care of myself missing 19	17/4.9	13/11.8	1.30 0.35–4.87	N.A.^2)^	N.A.^2)^

^1) ^odds ratio and 95% confidence interval.

^2) ^not applicable due to non-significance in bivariate analysis.

^3) ^13 of them were 'true missing', i.e. lived with children.

In group B, all diseases, all variables were significantly associated with sick listing in bivariate analyses (data not shown). In multivariate analysis, patient's report of severely limited work capacity significantly increased the chance of being sick listed for all patients as well as for patients with infectious or musculoskeletal diseases.

### GP and patient questionnaires

When all the significant variables from the GPs' and the patients' questionnaires from the previous regression analyses were introduced in a final regression model for all diseases the variables 'complaints entirely or primarily somatic', 'complaints severely limited the patient/me from occupational work' according to GP and to patient, and 'appointment for back, neck, arms/hands, leg/feet complaints' remained significantly related to being sick listed (Table 4). When restricting the analyses to patients with infectious diseases only the variable 'severely limited me from occupational work' according to the patient was significantly related to more frequent sick listing. For patients with musculoskeletal diseases 'severely limited from occupational work' according to GPs' and patients' questionnaires were the remaining significant variables. Adjusting for patient age, sex, native language, education, and professional status did not change the significance of the final regression results.

**Table 4:** Final multivariate analyses of the effects of significant variables in reports from GPs and patients on sick listing.

	All diseases (n = 409)		Infectious diseases (n = 144)		Musculoskeletal diseases (n = 103)	
	
	OR^1)^	99.5% CI	OR^1)^	99.5% CI	OR^1)^	99.5% CI
GPs' questionnaires:						
Complaints entirely or primarily somatic	0.16	0.04–0.61	0.03	0.001–1.72	0.096	0.003–3.41
Complaints severely limited the patient from occupational work	8.89	3.60–21.93	2.80	0.46–17.26	14.16	3.15–63.64
Complaints severely limited the patient from everyday pursuits	1.55	0.44–5.42	N.A^2)^.		N.A.^2)^	
Complaints severely limited the patient to fully take care of his/her children	N.A.^2)^		13.18	0.83–208.25	N.A.^2)^	
Patients' questionnaire:						
Exercising weekly or more often to sweatiness or breathlessness	N.A.^2)^		0.36	0.07–1.92	N.A.^2)^	
Sick-listed during last year	1.39	0.77–2.54	N.A.^2)^		1.93	0.66–5.61
Appointment for back, neck, arms//hands, legs/feet complaints	3.55	1.47–8.59	N.A.^2)^		N.A.^2)^	
Appointment for tiredness	2.16	0.55–8.58	N.A.^2)^		N.A.^2)^	
Complaints limited me from occupational work	7.01	2.69–18.29	6.35	1.59–25.38	7.91	1.69–36.95

^1) ^odds ratios (OR) and their 99.5% confidence intervals (CI).

^2) ^N.A. = not applicable due to non-significance in previous steps in multivariate analysis.

In Figure 2 the effects of various combinations of the patients' perceived work capacity and locomotor pain on the one hand and the GPs' assessment of the patients' work capacity and whether the disease was somatic on the other is presented. Ninety-six per cent of patients who claimed work incapacity and locomotor pain and where the physician confirmed the work incapacity and assessed the condition as not primarily somatic were sick listed. In contrast, only 2% of the patients with perceived work capacity and no locomotor pain and where the physician confirmed the work capacity and assessed the condition as somatic were sick-listed.

The degree of explanation of all the significant variables combined in the final multivariate models, for all, infectious and musculoskeletal diseases using r^2 ^was 36.1%, 10.9%, and 41.6%, and when using the ROC-method 78.4%, 41.3% and 77.2%, respectively.

### Effects of non-response

In 168 consultations GP data but no patient data and in 47 consultations patient data but no GP data were obtained (Figure 1). These patients were somewhat younger than the participants, mean age 38.3 years versus 41.4 years, the proportion of women was lower, 55.4% versus 64.3%, but the proportion who were sick listed was the same as for participants, 24.7% versus 24.5%. However, since none of these factors influenced the outcome, the effects of non-response seem to be neutral as far as the results are concerned.

## Discussion

For all diagnoses we found strong evidence that limited capacity to work, assessed by the patient as well as by the GP, was the most important factor when sickness certification was issued. The risk was increased with a factor nine if the GP and seven if the patient assessed that the complaints severely limited the patient's capacity to work. On the other hand, if neither the patient nor the physician assessed patient's capacity to work as severely limited, the risk of being sick listed was negligible (Figure 2). There are allegations that the patient's opinion rather than the GP's is decisive for the outcome sick leave or no sick leave [30,31]. Our findings point to a substantial degree of concordance between the opinions of two parties and suggest that the physician and patient share the same opinion on the patient's work ability.

We also found, that patients with pain in locomotor system had a threefold increased risk of being sick listed at the consultation. A strong reduction in the risk of being sick listed was seen when the GPs assessed the patient's complaints as being of primarily somatic origin. One explanation of this finding may be that a clear somatic cause of the patient's problem makes it easier for the GP to evaluate both the patient's functional impairments and potential and hence, in some cases, to abstain from sick listing the patient. When the physician sees a patient with locomotor system pain but no objective signs of disease it is more difficult to evaluate whether or not the patient is fit to work. In such a case it is likely that the physician will share their patients' negative views of their working capacity and sick list them.

As opposed to this view, our study shows that when patients had locomotor system complaints and the physicians could find justification for a musculoskeletal diagnosis there was no increased risk of being sick listed. In patients with a musculoskeletal diagnosis we found agreement between physicians and patients as to how much the patients' complaints prevented them from working. Actually, the GPs assessment of their patients working incapacity was almost twice as negative as the assessments of the patients themselves, the OR for a sick listing being 14.2 (physicians) versus 7.9 (patients).

The opposite held true for infectious diseases, where the patients' judgement of their own working incapacity increased the risk of being sick listed (OR 6,4), while the GP's opinion that the patient had limited work capacity did not result in sick listing. Most likely some patients with what they considered a not so alarming infectious symptoms resisted the GP's assessment and declined a sick note.

Since there is no definition of the disease concept, the field is open for the physician as well as the patient to use his/her own meaning of the concept. Also 'medical' is similar regarding lack of definition. Despite this, decisions on sick leave must be made, based on the best basis available. As long as a tool for estimating degree of functioning, like ICF [4], is not used, the individual understanding of the terms is to be used.

Our study results provide an important contribution to the view on the sick listing process. When GPs interpret their patients' complaints as being of somatic origin, there is a lesser inclination to put the patient on the sick list whether or not the working capacity of the patient is assessed as impaired. On the other hand, when a patient's complaint cannot be translated into somatic terms the GP, maybe in order not to endanger the patient-doctor relationship, may tend to sick list the patient although the objective findings of disease may be weak or absent. Such sick listing may be difficult to discontinue and may, in the long run, be detrimental for the patient.

Thus, psychiatric, psychosocial or social interference, or GP's suspicion of such interference on the illness, should be studied more specifically in the sick listing process. In our study the number of diagnoses referring to psychiatric problems was too small to be studied separately.

Although the degree of explanation in our material was lower for infectious diseases, a substantial part of sick leave certificates could be explained using our determinants. One third of the sick listing in the total group could be explained using the r^2 ^method. When we used the method measuring the area under the ROC curve, we found that more than three fourths of the sick listing could be explained by our determinants. This latter method is less biased by random variation and therefore a more efficient estimate of the degree of explanation. When investigating the impact of physician-related factors on sick listing, we found much lower degrees of explanation, with a maximum of between 8% with the r^2^method and 36% with the ROC method [10]. A Dutch study on employees of a university showed a stronger relation for health related aspects than for work related aspects to sick leave with a degree of explanation of 8% to 16% with the r^2 ^method [32]. Also, Shiels and Gabbay have shown that the diagnostic reason for the sickness episode explains more than 18% of the variance while clinician and general practice effects explain only 3.4% and 2.3% respectively [33].

The present article is based on data collected in a questionnaire study, intended to explore different aspects of the risk of being put on sick leave. Physician related factors were analysed in a previous paper [10]. In order to be able to gather the large quantity of data we needed, questionnaires were considered appropriate for the study.

The material in our study was collected in 1996. Since then sick leave rates have risen in Sweden and become a societal problem of dignity. Factors associated with the decision to write a sick note or not do not necessarily change over time or relate to sick leave rates, but if there has been a change in decision practice, factors related to this practice would rather be underestimated in our study. The year 1996 was just before the dramatic increase in Swedish sickness absence began, and can therefore be expected to constitute a baseline level when analysing reasons for granting sick leave. Since then psychiatric diagnoses have come to account for the majority of the increase in the Swedish sickness absence epidemic [34], likewise in Norway [35]. The non-somatic field of sicknesses is a complex one. Apart from purely psychiatric conditions, several societal and social conditions deserve attention. The need for better understanding of these matters is no less today than it was in 1996. We hope our study may serve as a reminder of this fact and stimulate further investigation of this area.

## Conclusion

The strongest indicators for sickness certification were patients' and GP's assessment of reduced work capacity. Concordance between physicians and patients on this assessment was conspicuous. When they agreed on work incapacity, 96 per cent of patients were sick listed, and when they agreed on work capacity, only 2 per cent were sick listed if assessment of degree of somatic disease and patient's presentation of symptoms were taken into consideration. When there are non-somatic causes of the work incapacity, the risk of being sick listed is increased.

## Competing interests

The author(s) declare that they have no competing interests.

## Authors' contributions

The study was designed by DA and GN. Statistical analyses were performed by DA and KS with the assistance of GN. GN, DA and KS drafted, revised and finally approved the manuscript.

## Pre-publication history

The pre-publication history for this paper can be accessed here:



## Supplementary Material

Additional file 1 Questionnaire to physicians about themselves. Click here for file

Additional file 2 Questionnaire to physicians about the consultation. Click here for file

Additional file 3 Questionnaire to the patients. Click here for file
