# Supplementary material for: How primary health care physicians make sick listing decisions: The impact of medical factors and functioning
Source: BMC Fam Pract. 2008 Jan 21;9:3. doi: 10.1186/1471-2296-9-3 (PMC2266928; doi:10.1186/1471-2296-9-3)
Supplement: Additional file 1 — Questionnaire to physicians about themselves. [file 1471-2296-9-3-S1.pdf]

Questionnaire on sick listing practices

I. Questions to physician.

1.	What is your sex?	male	☐
		female	☐
2.	What is your year of birth?	……..	
3.	Are you working in	private care	☐
		public care	☐
4.	What is your work position?	general practitioner	☐
		senior consultant	☐
		undergraduate physician	☐
		substitute	☐
5.	Are you a qualified specialist?	yes	☐
		no	☐
	If yes, in what speciality?	family medicine	☐
		other speciality	☐
		which? …………………..	
6.	How long have you been working in family medicine?	………. years	
7.	Do you have other experience of working life for more than six months than as a physician?	yes	☐
		no	☐
8.	Do you work	full time	☐
		part time	☐
		…....%	
9.	Do you normally (at least once a month on average) participate in any CME activity?	yes	☐
		no	☐

10. How often do you participate in the following further training activities?

	every week	once a month	every six months	never
"GP's days"	☐	☐	☐	☐
training sponsored by a pharmaceutical company	☐	☐	☐	☐
CME-group	☐	☐	☐	☐
other training …………………..	☐	☐	☐	☐

11. Do you have undergraduate training in social insurance medicine?

yes	☐
no	☐

12. In what form and how often do you participate in discussions on patient related problems

	every week	once a month	every six months	seldom/ never
physician's meetings in the practice	☐	☐	☐	☐
Balint group	☐	☐	☐	☐
CME-group	☐	☐	☐	☐
meeting with social worker	☐	☐	☐	☐
other discussion …………………	☐	☐	☐	☐

13. I have contact with the social insurance office on average

every week	☐
every month	☐
every six months	☐
never	☐

14. My contacts with the social insurance office are

in writing	☐
by telephone	☐
mini-rehabilitation meeting	☐
other rehabilitation meeting	☐
physicians' meetings with social insurance officers	☐
other contact ……………………………………..	☐

Consider the following statements. *(Mark your opinion by a cross on the scale)*
(SIO = Social Insurance Office)

15. At present, the function of the SIO is to assist the sick person in returning to work as quickly as possible.

    Disagree |____| Agree

16. The SIO's participation in rehabilitation work is mainly formal.

    Disagree |____| Agree

17. The SIO's participation is an asset in rehabilitation work.

    Disagree |____| Agree

18. The physician has the greatest influence on rehabilitation work.

    Disagree |____| Agree

19. The SIO has the greatest influence on rehabilitation work.

    Disagree |____| Agree

20. The employer has the greatest influence on rehabilitation work.

    Disagree |____| Agree

21. The patient has the greatest influence on rehabilitation work.

    Disagree |____| Agree

22. Patient, physician, SIO and employer can find a shared goal for the rehabilitation work through discussions.

    Disagree |____| Agree

23. SIO officers have good knowledge of work in various occupations.

    Disagree |____| Agree

24. The SIO's regulations are an obstacle to rehabilitation work.

    Disagree |____| Agree

25. The SIO's regulations are an asset in rehabilitation work.

    Disagree |____| Agree

26. It is a good thing that social insurance officers make home visits to ensure that there is no cheating with sickness certification.

Disagree |____| Agree

27. The SIO's advising physicians see to the best interests of the patient.

Disagree |____| Agree

28. The SIO's advising physicians act on the basis of overly detailed regulations.

Disagree |____| Agree

29. Personal problems and worries may influence the pain a patient has from a shoulder complaint.

Disagree |____| Agree

30. Patients should be allowed to be on sick leave when they are tired and depressed.

Disagree |____| Agree

31. Many people report sick without being ill.

Disagree |____| Agree

32. Many people are on long-term sick leave without being ill.

Disagree |____| Agree

33. If a person gets on well at his/her job, he/she will want to be on sick leave for as short a time as possible.

Disagree |____| Agree

34. Sick leave benefits encourage unnecessary sick listing.

Disagree |____| Agree

35. If a patient asks to be sick listed, the physician has no choice but to sick list him/her.

Disagree |____| Agree

36. A physician's work situation affects his/her sick listing practices.

Disagree |____| Agree

37. Physicians have good knowledge of work in various occupations.

Disagree |____| Agree

38. The physician's decision on sick listing is sometimes in conflict with the patient's opinion.

Disagree |______________________________________| Agree

39. I sometimes sick list a patient even when it is not medically justified.

Disagree |______________________________________| Agree

40. I sometimes sick list a patient for social reasons.

Disagree |______________________________________| Agree

41. The social insurance system does not encourage job seeking.

Disagree |______________________________________| Agree

42. Today's social insurance system is good.

Disagree |______________________________________| Agree

43. Most employers are eager to have employees with a disease or a handicap at the workplace.

Disagree |______________________________________| Agree

44. Most employers have a positive attitude towards part time sick leave.

Disagree |______________________________________| Agree

45. Being unemployed for a long time is harmful to one's health.

Disagree |______________________________________| Agree

46. Being on sick leave for a long time is harmful to one's health.

Disagree |______________________________________| Agree

47. My general health is good.

Disagree |______________________________________| Agree

48. I get on well at my job.

Disagree |______________________________________| Agree

49. My knowledge of the Social Insurance System is insufficient.

Disagree |______________________________________| Agree

50. The number of patients on my list in 1995 was ………
(last known data)

51. At this surgery our staff of physicians is
- much too large ☐
- somewhat large ☐
- adequate ☐
- somewhat small ☐
- much too small ☐

52. A this surgery
- patients have open access to the surgery ☐
- patients have open telephone access to the physician every day ☐
- appointments are mostly planned directly by the physician ☐
- appointments are mostly planned by the nurse ☐
- appointments are mostly planned in writing ☐

Thank you for your participation!
