# Supplementary material for: How primary health care physicians make sick listing decisions: The impact of medical factors and functioning
Source: BMC Fam Pract. 2008 Jan 21;9:3. doi: 10.1186/1471-2296-9-3 (PMC2266928; doi:10.1186/1471-2296-9-3)
Supplement: Additional file 2 — Questionnaire to physicians about the consultation. [file 1471-2296-9-3-S2.pdf]

II. Questions on the patient and this consultation

The following questions should be answered for every patient who accepts a questionnaire. The patient's and your form should have the same number in order to be cross-linked. Neither your identity, nor the patient's identity will be revealed.

53. The patient is a
    - man ☐
    - woman ☐

54. Patient's year of birth ……..

55. The patient's family doctor is
    - myself ☐
    - another GP at the same health care centre ☐
    - a GP at another health care centre ☐
    - a private GP ☐
    - unknown ☐
    - the patient has no family doctor ☐

56. I have seen the patient before
    - many times ☐
    - once ☐
    - never ☐

57. The visit was a
    - first visit for this complaint (no visit in the last 12 months) ☐
    - follow-up appointment ☐

58. The consultation was a
    - planned consultation (= booked no later than 24 hours prior to the consultation) ☐
    - emergency consultation after telephone contact with the physician ☐
    - other emergency consultation ☐

59. I assess the patient's complaint as
    - entirely somatic ☐
    - primarily somatic ☐
    - equally somatic and psychiatric ☐
    - primarily psychiatric ☐
    - entirely psychiatric ☐

60. I estimate that the patient will have recovered within
    - one week ☐
    - one month ☐
    - three months ☐
    - more than three months ☐
    - recovery time cannot be estimated ☐

61. My diagnosis is
- very certain ☐
- fairly certain ☐
- neither certain nor uncertain ☐

62. The patient's disease/complaint is classified in the following group (International Classification of Diseases 9th revision).
If more than one diagnosis is applicable, rank them with 1, 2 and 3 according to your judgement of their importance for the present consultation.

- I Infectious diseases ☐
- II Tumours ☐
- III Endocrine diseases ☐
- IV Blood diseases ☐
- V Psychiatric diseases ☐
- VI Diseases in nervous system ☐
- VII Diseases in circulatory system ☐
- VIII Diseases in respiratory system ☐
- IX Diseases in digestive system ☐
- X Diseases in uro-genital system ☐
- XI Complications to pregnancy and childbearing ☐
- XII Skin disorders ☐
- XIII Diseases in musculoskeletal system ☐
- XIV Symptoms and unspecified cases ☐
- XV Trauma and poisonings ☐
- None of the groups above ☐

63. I assess that the patient's complaints will restrain her/him from

	not at all	moderately	severely
occupational work	☐	☐	☐
everyday pursuits	☐	☐	☐
taking care of her/his children	☐	☐	☐
activities of daily living	☐	☐	☐
physical leisure time activities	☐	☐	☐
social leisure time activities	☐	☐	☐
intellectual leisure time activities	☐	☐	☐
sleeping at night	☐	☐	☐

64. I assess that as regards the decision about sick leave/sick listing the patient and I were
- completely in agreement ☐
- mainly in agreement ☐
- neither in agreement nor not in agreement ☐
- not completely in agreement ☐
- seriously in disagreement ☐
- sick leave/sick listing did not come up ☐

Questions 65-68 should be answered ***ONLY*** *if you sick listed the patient or gave the patient a recommendation to report sick:*

65. Did you recommend the patient to report sick
    - yes ☐
    - no ☐

66. How many days did you report the patient sick on the sickness certificate ……. days

67. Degree of sickness certification
    - 100% ☐
    - 75% ☐
    - 50% ☐
    - 25% ☐

68. You have certified the patient sick or recommended him/her to stay at home for a certain number of days. Estimate how long you think the length of the sick leave period will be
    - 1-3 days ☐
    - 4-7 days ☐
    - 8-14 days ☐
    - 15-29 days ☐
    - 30-89 days ☐
    - more than 90 days ☐

Thank you for your participation!
