# Supplementary material for: How primary health care physicians make sick listing decisions: The impact of medical factors and functioning
Source: BMC Fam Pract. 2008 Jan 21;9:3. doi: 10.1186/1471-2296-9-3 (PMC2266928; doi:10.1186/1471-2296-9-3)
Supplement: Additional file 3 — Questionnaire to the patients. [file 1471-2296-9-3-S3.pdf]

Questionnaire on the consultation with the doctor and sick listing

1. What is your year of birth? …………

2. What is your sex?
   - male ☐
   - female ☐

3. What is your education?
   - comprehensive school ☐
   - secondary school ☐
   - continuation/vocational school ☐
   - upper secondary school ☐
   - university ☐

4. Is Swedish your native language?
   - yes ☐
   - no, my native language is ……………………………..…

Questions about your work situation:

5. What was your employment status at the time of the consultation?
   - permanently employed ☐
   - temporarily employed ☐
   - unemployed ☐
   - self-employed ☐
   - home-worker ☐
   - student ☐
   - on parental leave ☐
   - other ………………………..... ☐

6. What was your occupation at the time of the consultation?

   ………………………………………

7. How much did you work/study at the time of the consultation?
   - full time or more ☐
   - more than half time but not full time ☐
   - half time ☐
   - less than half time ☐

8. *If you were unemployed*:

   For how long had you been unemployed?
   - less than one month ☐
   - one month or more, but less than six months ☐
   - more than six months but less than one year ☐
   - more than one year ☐

Questions about your job

*If you have been unemployed for **more than one year**, go on to question 29.*
If you are working or are unemployed since less than one year, please answer the questions relative to your last job.

		always	mostly	some-times	seldom	never
9.	Can you influence the pace at which you work?	☐	☐	☐	☐	☐
10.	Can you decide *what* to do during the working day?	☐	☐	☐	☐	☐
11.	Can you influence *how* you carry out your work?	☐	☐	☐	☐	☐
12.	Do you find your job stimulating?	☐	☐	☐	☐	☐
13.	Do you think your job is considered important in society?	☐	☐	☐	☐	☐
14.	Can you confer with your co-workers on difficult matters?	☐	☐	☐	☐	☐
15.	Can you get support from your supervisor/manager on difficult matters?	☐	☐	☐	☐	☐
16.	Is being together with your co-workers important to you?	☐	☐	☐	☐	☐
17.	Is there a good atmosphere at your workplace?	☐	☐	☐	☐	☐
18.	Does your work demand a high level of skill or expertise?	☐	☐	☐	☐	☐
19.	Does your job require you to take the initiative?	☐	☐	☐	☐	☐
20.	Do you get your work done on time?	☐	☐	☐	☐	☐
21.	Do you worry about being injured at or becoming ill from your work?	☐	☐	☐	☐	☐
22.	Do you worry about changes in your work situation (dismissal, change of organisation, etc.)?	☐	☐	☐	☐	☐
23.	Can you alternate between easy and difficult tasks?	☐	☐	☐	☐	☐
24.	Do you have the possibility of learning new things through your job?	☐	☐	☐	☐	☐
25.	Do you have to do the same thing over and over again?	☐	☐	☐	☐	☐
26.	Does your work often involve conflicting demands?	☐	☐	☐	☐	☐

27. Does your job make you feel *physically* exhausted? ☐ ☐ ☐ ☐ ☐

28. Does your job make you feel *mentally* exhausted? ☐ ☐ ☐ ☐ ☐

On the scales below, try to estimate your agreement or disagreement with the various statements. The scales range from “disagree” to “agree”. Mark your opinion by a cross on the line somewhere along the scale.

29. I get on well at my job.

Disagree |____| Agree

30. Most employers are eager to have employees with a disease or a handicap at the workplace.

Disagree |____| Agree

31. If a person gets on well at his/her job, he/she wants to be on sick leave as short a time as possible.

Disagree |____| Agree

32. Physicians have good knowledge of work in various occupations.

Disagree |____| Agree

33. Being unemployed for a long time is harmful to one’s health.

Disagree |____| Agree

34. Most employers have a positive attitude towards part time sick leave.

Disagree |____| Agree

Questions about your health:

35. How often were you on sick leave (with or without a doctor’s certificate) during the last year before the consultation?

- not at all ☐
- 1-3 times ☐
- 4-6 times ☐
- more than 6 times ☐

36. What was your total time on sick leave the year before the consultation?

- one week or less ☐
- more than one week but less than one month ☐
- one month or more, but less than three months ☐
- three months or more ☐

37. How do you assess your general health status?

- I am mostly healthy and feel well ☐
- I have one or more diseases, but I feel well most of the time ☐
- I have no known diseases, but I feel fairly unwell ☐
- I have one or more diseases and I seldom or never feel well ☐

How do you think diseases and health problems should be handled?

Mark a cross on the line for every statement regarding how you feel it corresponds to your opinion

38. When you have a cold, it is important to stay at home in order not to contaminate your workmates.

Disagree |_______________________________________| Agree

39. You should be allowed to be on sick leave when you are tired and depressed.

Disagree |_______________________________________| Agree

40. It is a good thing that social insurance officers make home visits to ensure that there is no cheating with sickness certification.

Disagree |_______________________________________| Agree

41. Being on sick leave for a long time is harmful to one's health..

Disagree |_______________________________________| Agree

42. If you do not have a fever you do not need to be on sick leave for a common cold.

Disagree |_______________________________________| Agree

43. You recover more quickly from illness if you rest and take it easy.

Disagree |_______________________________________| Agree

44. Family situation – *tick the alternative that is most relevant to your situation*

I live: alone ☐
with parent(s) ☐
with wife/husband/live-in partner ☐
with wife/husband/live-in partner and (number) ….. children ☐
with (number) ….. children ☐

Questions about your opportunities for using your leisure time for activities making you feel good:

45. Which of these activities do you do in your leisure time?
(One cross in each row)

		seldom or never	once a month	once a week	two or more times a week	daily
a.	I exercise at least 20 minutes at a pace that makes me sweaty and out of breath.	☐	☐	☐	☐	☐
b.	I exercise at least 20 minutes at a quick pace but without being sweaty or out of breath.	☐	☐	☐	☐	☐
c.	I am active outdoors at a calm pace (walking, garden work, hunting, fishing, etc.).	☐	☐	☐	☐	☐
d.	I spend time with my family/children.	☐	☐	☐	☐	☐
e.	I spend time with other people than family.	☐	☐	☐	☐	☐
f.	I read, listen to music, play computer games, look at TV/video, etc.	☐	☐	☐	☐	☐
g.	I do hobby activities at home (needlework, woodwork, repair work, etc.).	☐	☐	☐	☐	☐
h.	I participate in group activities outside the home (study circle, hobby group, music group, etc.).	☐	☐	☐	☐	☐
i.	I participate in club or church or trade union activities or similar.	☐	☐	☐	☐	☐
j.	I go to the cinema, theatre, dancing, football games or similar.	☐	☐	☐	☐	☐
k.	other …………………………………………..	☐	☐	☐	☐	☐

Questions about the present consultation:

46. Why did you contact the doctor? *(More than one cross permitted)*

For:

complaints/pain in back, neck, arms/hands, legs/feet	☐
complaints/pain in abdomen/digestion	☐
anxiety, nervousness, depression or insomnia	☐
complaints/pain in chest or heart	☐
complaints/pain in respiratory tract	☐
complaints/pain in urinary tract	☐
headache, dizziness or balance problems	☐
rash, eczema, or other skin eruptions	☐
tiredness	☐
fever, possible infection	☐
injuries from accidents or similar (i.e. wounds, swellings, burns)	☐
complaints/pain in genital tract, menstrual disorders	☐
complaints/pain in connection with eyes/vision or ears/hearing	☐
other,	☐

specify ………………………………………………………

47. Did these complaints/pains keep you from
(One cross in each row)

	not at all	moderately	very much
performing everyday pursuits at home	☐	☐	☐
seeing friends	☐	☐	☐
taking care of yourself	☐	☐	☐
performing your general leisure time activities	☐	☐	☐
sleeping	☐	☐	☐

48. Did the complaints/pains keep you from
(One cross in each row)

	not at all	moderately	very much	not applicable
occupational work	☐	☐	☐	☐
spending time with your family	☐	☐	☐	☐
taking care of your children	☐	☐	☐	☐
other ……………………………		☐	☐	

49. Had you had previous consultations with the physician you saw this time?
- no, I had never seen this physician before ☐
- yes, occasionally ☐
- yes, many times ☐

How important was it to you to get help with the matters below at specifically this appointment? *(One cross on the line for each statement!)*

50. To find out the cause of the complaints.

Very important |______________________________| Not important at all

51. To find out if it was serious

Very important |______________________________| Not important at all

52. To get medication to alleviate/cure the condition

Very important |______________________________| Not important at all

53. To have tests

Very important |______________________________| Not important at all

54. To get a referral to a specialist, x-ray, etc.

Very important |______________________________| Not important at all

55. To be sick listed

Very important |______________________________| Not important at all

56. To get a referral for treatment (to a physiotherapist, psychologist, etc.)

Very important |______________________________| Not important at all

57. To have the opportunity, in dialogue with the doctor, to get advice on what could be done to recover

Very important |______________________________| Not important at all

58. Other ……………………………………………………….

Very important |______________________________| Not important at all

59. How much did you want to be sick listed?

- not at all ☐
- ¼-time ☐
- half time ☐
- ¾-time ☐
- full time ☐
- could not decide ☐

60. If you did **NOT** want to be sick listed by the doctor: why?

- my work capacity was not much affected ☐
- I could not afford to be on sick leave ☐
- sickness absence causes great problems at my work ☐
- not needed, I was free from work for another reason ☐
- I had reported sick but did not need a certificate ☐
- other reasons ……………………………………… ☐

61. How long did you want to be on sick leave?
(If you did NOT want to be on sick leave, go on to question 62)

- 1-3 days ☐
- 4-7 days ☐
- 8-14 days ☐
- 15-29 days ☐
- 30 days or more ☐
- could not decide ☐

62. Would it have been necessary to be sick listed if you had another type of occupation?

- yes ☐
- no ☐
- do not know ☐

63. Was sick listing brought up during the consultation?

- no ☐
- yes, by the doctor ☐
- yes, by me ☐
- yes, but I do not remember by whom ☐

64. Were you sick listed?

- no ☐
- yes, ¼-tim ☐
- yes, half time ☐
- yes ¾-time ☐
- yes, full time ☐

65. For how long were you sick listed?

- 1-3 days ☐
- 4-7 days ☐
- 8-14 days ☐
- 15-29 days ☐
- 30 days or more ☐

66. How was the consultation carried out?

	yes	no
the doctor asked me questions	☐	☐
the doctor examined me	☐	☐
the doctor or a nurse took tests	☐	☐
the doctor consulted someone else about my complaints	☐	☐

67. What did the doctor get to know about your job situation during the consultation?
(Tick one box for each question)

The doctor asked/I told the doctor:	yes	no	the doctor already knew
where I work	☐	☐	☐
what I do at my job	☐	☐	☐
how I perform my work tasks	☐	☐	☐
how much I work (full time, part time)	☐	☐	☐
how I get to and from my work	☐	☐	☐
how the complaints would influence my work capacity	☐	☐	☐

68. Did the doctor, during the consultation, get to know something else about your situation that you think is important to how you felt?
(Tick whatever boxes are relevant)

- matters concerning my job situation, for example disagreements, heavy workload, problem with workmates or manager, risk for unemployment ☐
- childcare problems ☐
- financial problems ☐
- family problems ☐
- illness or need for care in the family ☐
- recent death in the family or a close relative ☐
- change of housing, place or country ☐
- other, describe shortly…………………… ………… ☐

69. How were you informed of the results of the examination at the consultation?
Tick the most relevant alternative. ***Only one tick!***

- the doctor and I talked about my complaints ☐
- I asked the doctor and he/she answered ☐
- I received no information ☐

70. Did you get a referral for another examination or treatment because of your complaint/pain

- yes ☐
- no ☐

for what kind of treatment/examination? ………………………………….

71. What measures did the doctor suggest you to take in order to recover?

- I received a prescription or was recommended an over the counter drug ☐
- I got advice on what to eat or drink ☐
- I got advice on exercise ☐
- I got advice on smoking ☐
- I got advice on what to do or avoid doing ☐
- other ……………………………………………. ☐

72. Were other measures suggested because of your health status?

- the doctor asked if he/she could contact the social insurance office ☐
- I got advice on contact with work ☐
- another visit was scheduled ☐
- a telephone appointment was scheduled ☐
- other ………………………………………………… ☐

73. What do you think was important for the doctor's decision to sick list you or not to sick list you?
(Tick one box in each row)

	very great importance	great impor-tance	some impor-tance	no impor-tance	do not know
the doctor's assessment from examination and tests	☐	☐	☐	☐	☐
the doctor's assessment of my health status based on my medical history	☐	☐	☐	☐	☐
the doctor's assessment of my ability to cope with my work	☐	☐	☐	☐	☐

74. How did you experience your consultation at the surgery?

	totally	mainly	neither nor	not very much	not at all
I was well treated by the staff at the surgery	☐	☐	☐	☐	☐
waiting time was reasonable	☐	☐	☐	☐	☐

75. How did you experience the doctor during the consultation?
Tick one box in each line!

The doctor was:	completely	mostly	fairly	not at all	do not know
competent	☐	☐	☐	☐	☐
calm	☐	☐	☐	☐	☐
sensitive	☐	☐	☐	☐	☐
experienced	☐	☐	☐	☐	☐
thorough	☐	☐	☐	☐	☐
understanding	☐	☐	☐	☐	☐
committed	☐	☐	☐	☐	☐
proper	☐	☐	☐	☐	☐

76. How do you regard the results of the consultation?
- I am completely satisfied ☐
- I am rather satisfied ☐
- I am neither satisfied nor dissatisfied ☐
- I am rather dissatisfied ☐
- I am very dissatisfied ☐

77. If you have any comments on our questionnaire and the questions, please write them here or continue on a separate piece of paper.

………………………………………………………………………………

………………………………………………………………………………

………………………………………………………………………………

………………………………………………………………………………

………………………………………………………………………………

………………………………………………………………………………

………………………………………………………………………………

………………………………………………………………………………

Thank you for your participation!
